# Hydrolytic activity determination of Tail Tubular Protein A of *Klebsiella pneumoniae* bacteriophages towards saccharide substrates

**DOI:** 10.1038/s41598-017-18096-1

**Published:** 2017-12-22

**Authors:** Ewa Brzozowska, Anna Pyra, Krzysztof Pawlik, Monika Janik, Sabina Górska, Natalia Urbańska, Zuzanna Drulis-Kawa, Andrzej Gamian

**Affiliations:** 1 Ludwik Hirszfeld Institute of Immunology and Experimental Therapy, Polish Academy of Sciences, 12 R. Weigl, 53–114 Wroclaw, Poland; 2 Faculty of Chemistry, Department of Crystallography, University of Wroclaw, 14 F. Joliot-Curie, 50–383 Wroclaw, Poland; 3 Department of Biological Science, University of Wroclaw, 35 Kuznicza, 50–138 Wroclaw, Poland; 4 Department of Pathogen Biology and Immunology, Institute of Genetics and Microbiology, University of Wroclaw, 63–77 Przybyszewskiego, 51–148 Wroclaw, Poland; 5 Wroclaw Research Center EIT+, 147 Stablowicka, 54–066 Wroclaw, Poland

## Abstract

In this paper, the enzymatic activity, substrate specificity and antibiofilm feature of bacteriophage dual-function tail proteins are presented. So far, tail tubular proteins A–TTPAgp31 and TTPAgp44-have been considered as structural proteins of *Klebsiella pneumoniae* bacteriophages KP32 and KP34, respectively. Our results show that TTPAgp31 is able to hydrolyze maltose as well as Red-starch. The activity of 1 µM of the protein was calculated as 47.6 milli-Units/assay relating to the α-amylase activity. It degrades capsular polysaccharides (cPS), slime polysaccharides (sPS) and lipopolysaccharide (LPS) of *K*. *pneumoniae* PCM 2713 and shows antibiofilm reactivity towards *S*. *aureus* PCM 519 and *E*. *faecalis* PCM 2673. TTPAgp44 hydrolyses trehalose and cPS of *E*. *faecium* PCM 1859. TTPAgp44′s activity was also observed in the antibiofilm test against *P*. *aeruginosa* PCM 2710 and *B*. *subtilis* PCM 2021. TTPAgp31 has been identified as α-1,4-glucosidase whereas, TTPAgp44 exhibits trehalase-like activity. Both proteins contain aspartate and glutamate residues in the β-stranded region which are essential for catalytic activity of glycoside hydrolases. The significant novelty of our results is that for the first time the bacteriophage tubular proteins are described as the unique enzymes displaying no similarity to any known phage hydrolases. They can be used as antibacterial agents directed against bacterial strains producing exopolysaccharides and forming a biofilm.

## Introduction

Biofilm forming pathogens are a widespread problem especially in medicine, food technologies, pharmaceutical and biohydrometallurgical industry. In the food industry, the biofilm-forming bacteria cause spoilage of the products due to the colonization of industrial equipment surface and food, leading to infections among consumers. Similarly, biofilm formed in the interior of water supply pipes results in the rise of human health risk and additionally contributes to metal corrosion and reduction of water flow. Biofilm formation on medical implant devices such as catheters and mechanical heart valves, is also a medical problem. All these aspects determine the widespread interest among researchers in the biofilm formation and functioning. In recent years attention has been paid to methods of recognizing and combating biofilm-forming microbial populations^1–3^.

Human pathogens associated with biofilm development include species of: *Staphylococcus aureus*, *Staphylococcus epidermidis*, *Enterococcus faecalis*, *Enterococcus faecium*, *Escherichia coli*, *Proteus mirabilis*, *Klebsiella pneumoniae*, *Streptococcus viridans*, *Pseudomonas aeruginosa* and *Acinetobacter* spp. These bacteria are the cause of chronic and recurrent infections (over 60% cases), particularly in the hospital environment^4^.

*Klebsiella pneumoniae* strains belonging to the *Enterobacteriaceae* family are widely distributed in the environment^5^. The genus *Klebsiella* comprises bacteria that cause different types of healthcare-associated infections, including pneumonia, bloodstream, urinary and intestinal tract infections, wound or surgical site infections, meningitis and neonatal sepsis^6^. An important virulence factor and a defense barrier of *K*. *pneumoniae* is polysaccharide layer, referred to as a capsule^7^. Inhibition of capsular polysaccharides (cPSs) synthesis enhances the phagocytosis of encapsulated bacteria by human neutrophils and consequently their elimination^8^. It has been shown that the larger the capsule produced, the more pathogenic the strain is^7^. Encapsulated *K*. *pneumoniae* also produces large quantities of cell-free PS (slime PS) which is secreted into the external environment and is another important virulence factor contributing to pathogenicity^8^. PSs are also a crucial structural component of bacterial biofilm, which promotes attachment to the surface and protects microorganisms from antimicrobials^9^. Therapeutic agents destroying bacterial capsular and/or slime PSs provide a useful adjunct to therapy against infections caused by encapsulated bacteria, especially if they are not chemically synthesized antibiotics^7^.

Bacteriophages are a promising alternative for this purpose. Their antibacterial efficiency is well documented^10–14^. However, they are still classified as high-risk medications due to the release of endotoxin into the bloodstream after bacterial cell lysis^15^. Therefore, the use of molecules such as hydrolyzing enzymes of phage origin provides better control over the therapeutic effects on the organism^16,17^. Some of the hydrolyzing enzymes are localized on bacteriophage tail. According to recent scientific data, 120 of the 160 different phage hydrolyses are coded in the same open reading frame as structural proteins (tail and fibers proteins). Therefore, they have been also considered as structural proteins, not as enzymes^18^.

Previously, we have reported the preliminary results of biological activity of the tail tubular protein A of the phage KP32 tail which is a product of a gene number 31 (TTPAgp31)^19^. This protein we called the dual-function protein due to its structural and hydrolytic features related to binding and hydrolysis of EPS obtained from *K*. *pneumoniae* PCM 2713.

In this paper, we show the results of the next biological tests of TTPAgp31 and for the first time we demonstrate the results of biological activity of the protein named TTPAgp44 which comes from KP34 bacteriophage tail and it is a product of a gene number 44.

Here we present our efforts to determine the substrate specificity of two TTPAs using versatile chromogenic polysaccharide substrates with well-defined chemical structures. We also demonstrate screening tests of TTPAs mentioned above displaying antibiofilm effect which is due to hydrolytic activity towards extracellular polysaccharides.

## Results

### Cloning, genes expression, proteins purification and analysis

Tail tubular proteins A from *K*. *pneumoniae* bacteriophages were selected for study as potential secondary adhesive proteins (adhesins), which bind to receptors localized on the bacterial cell walls. These proteins belong to a group of homologous proteins present in a wide range of bacteriophages specific to several strains of Gram-negative and Gram-positive bacterial species. Both TTPAs are built of 192 amino acid residues however, their isoelectric points (Ip) are quite different, for TTPAgp31 the Ip is 4.26 and for TTPAgp44 is 8.13. BLAST amino-acid sequence analysis showed that the homology between them is about 21.5%, on the other hand, analysis using Phyre 2 server^20^ revealed that 88% of TTPAgp44 amino acids share the same structure as TTPAgp31. As we reported previously^19^, in the case of TTPAgp31 high amino acid sequence similarity (about 64%) to the gatekeeper protein (gp11) of T7 bacteriophage has been found, while TTPAgp44 is similar to gp11 only at 29%. Further analysis using a HHPred tool^21^ showed that both TTPAs share peptidoglycan hydrolase domain with probability about 40%.

**Figure 1:**
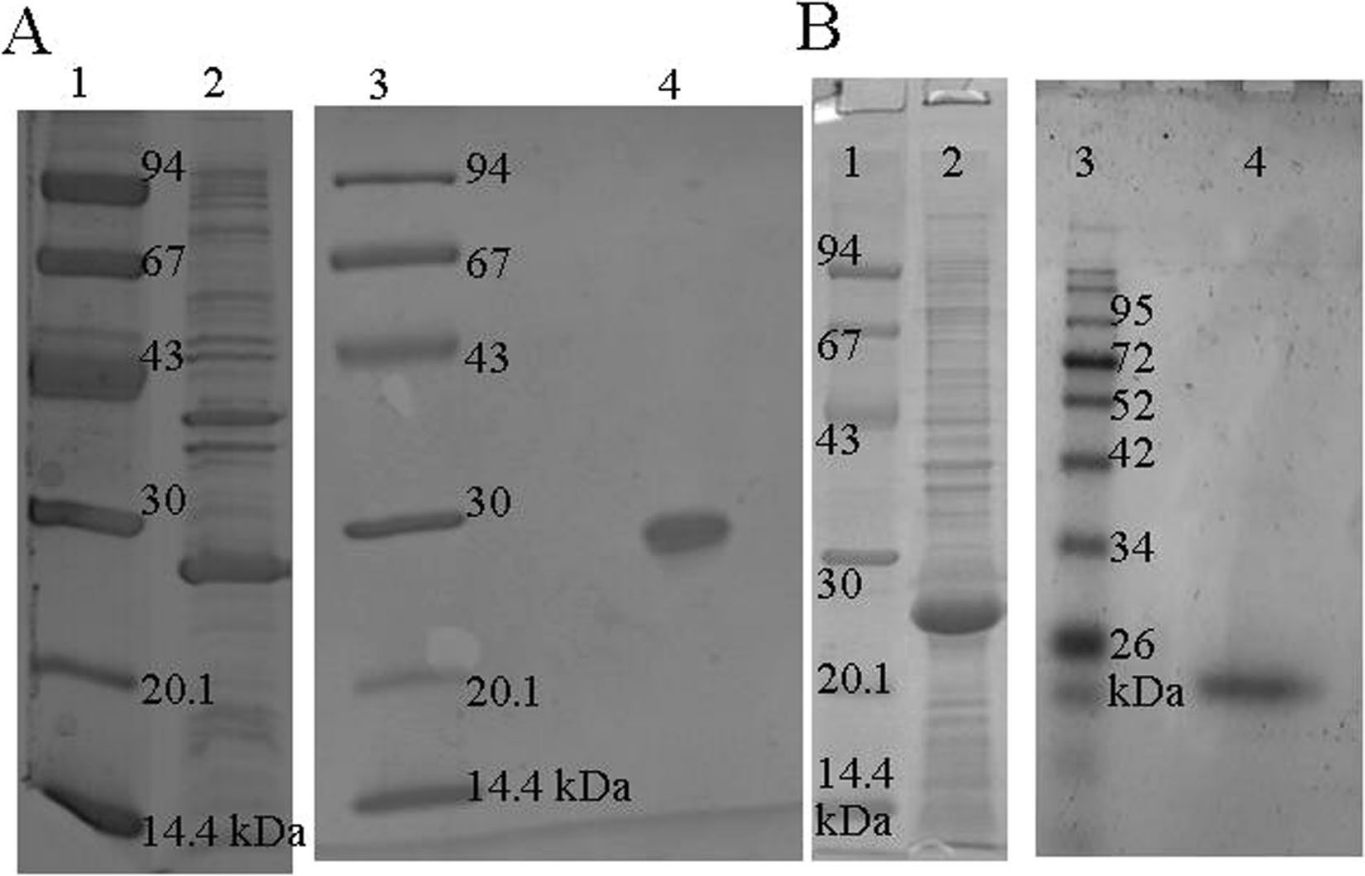
Electrophoretic analysis of TTPAs proteins expressed in *E*. *coli* DE3plysS in 12.5% SDS-PAGE.TTPAgp31 before (line 2) and after (line 4) purification – The results have been previously reported by Pyra *et al*.^19^; 1) Low molecular mass standard (Bio-Rad), B) TTPAgp44 before (line 2) and after (line 3) purification on Ni^2+^-affinity chromatography, (1) molecular mass standard (Thermo Scientific).

The results of the protein expression and purification are presented in Fig. 1. As a result: 25 mg of TTPAgp31 and 20 mg of TTAgp44 from one liter of bacterial culture was obtained. The purified proteins were analyzed by SDS-PAGE. The electrophoretic analysis of TTPAgp31 (Fig. 1A) have been previously reported by Pyra *et al*.^19^ and the SDS-PAGE results for TTPAgp44 (Fig. 1B) are presented here for the first time.

### Agar overlay test

**Figure 2:**
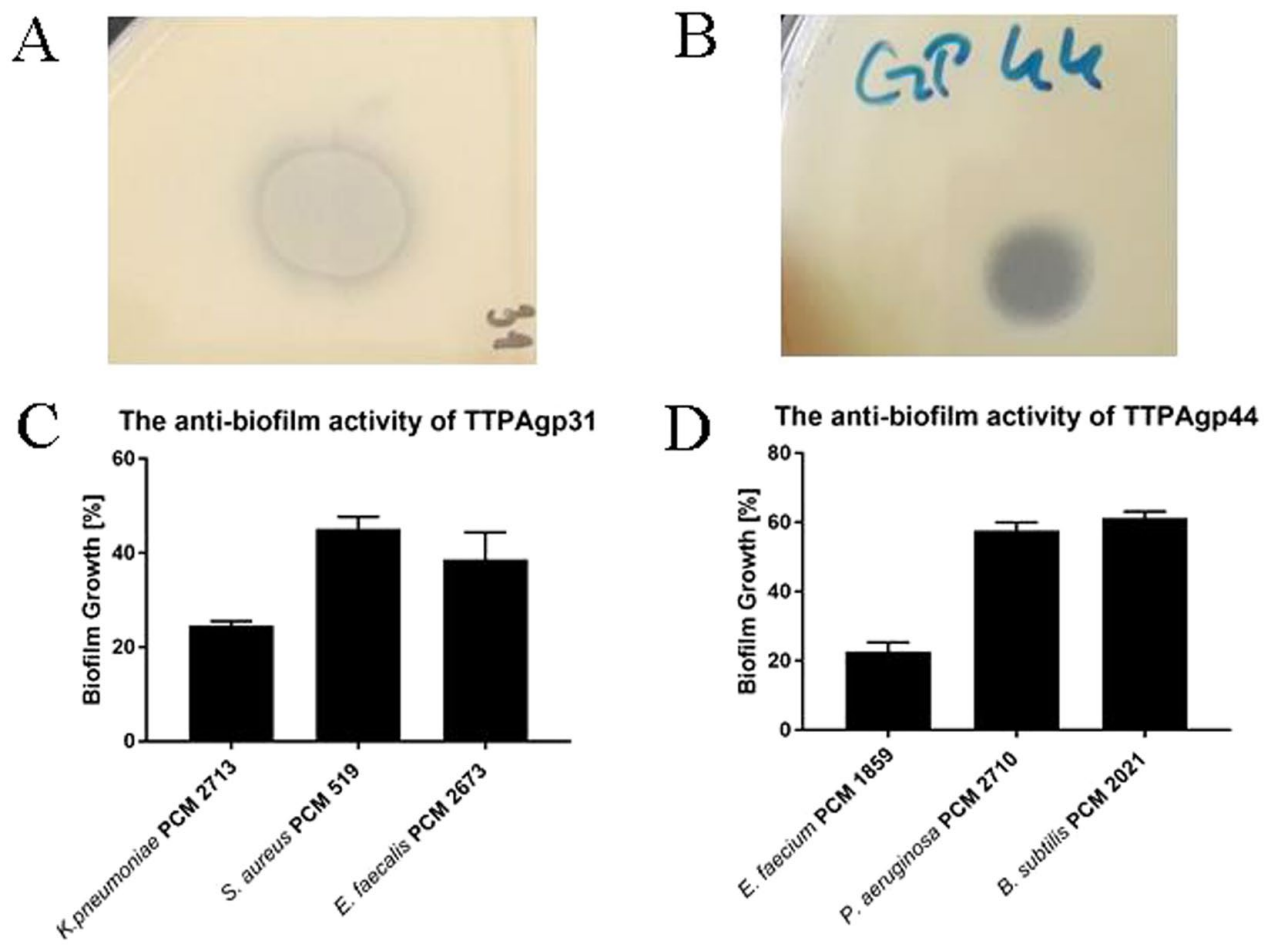
The activity of TTPAs towards bacterial strains. **(A)** overlay agar test towards *Klebsiella pneumoniae* PCM 2713 and **(B)** *Enteroccocus faecium* PCM 1859 lawn. In both cases 10 µl of 2 mM of the proteins were spotted on mature bacterial lawns and incubated at 37 °C overnight. **(C)** Antibiofilm activity of TTPAgp31 and TTPAgp44 (0.47 µM) **(D)** detected by TTC assay. The amount of biofilm formed after 20 h of incubation was estimated. Absorbance was measured at 540 nm following incubation with TTC (0.1%). All results are presented as averages of results from three independent replicates in three parallel trials. Error bars represent the means, standard deviations (GraphPad Prism version 5.00 for Windows, GraphPad Software, San Diego California USA, (www.graphpad.com)). The authors have reused the Fig. 2A previously reported in^19^.

Initial screening tests that assess the enzymatic activity of the phage proteins were agar spot assays on plates with mature bacterial lawns. Eighteen selected strains both from Gram-negative and positive group have been tested. TTPAgp31 showed its ability to create activity zone on *K*. *pneumoniae* PCM 2713, *S*. *aureus* PCM 519 and *E*. *faecalis* PCM 2673 lawn in the same test conditions. TTPAgp44 was active against thermoresistant *E*. *faecium* PCM 1859, *P*. *aeruginosa* PCM 2710 and *B*. *subtilis* PCM 2021. The activity zones were translucent halo (not clear plaques) and all sensitive strains have been able to produce slime as well as to form a biofilm. Moreover, strains: PCM 2673, 2710 and 2713 have been documented as clinical human isolates. Further analysis showed that TTPAs can also reduce bacterial biomasses of above strains. It was shown that 0.47 µM of TTPAgp31 can reduce about 80%, 60% and 50% of *K*. *pneumoniae*, *E*. *faecalis* and *S*. *aureus* strains’ biomass, respectively after one-day biofilm formation assay. While 0.47 µM of TTPAgp44 reduces about 80% of *E*. *faecium* biomass and about 40% of *P*. *aeruginosa* and *B*. *subtilis* strains; biomass in the same test conditions. The antibiofilm activity of the two phage proteins is shown in Fig. 2. To compare the spots created by both TTPAs, we have reused the figure of TTPAgp31 activity on agar plate (Fig. 2A) presented previously in^19^.

### Hydrolytic activity

**Figure 3:**
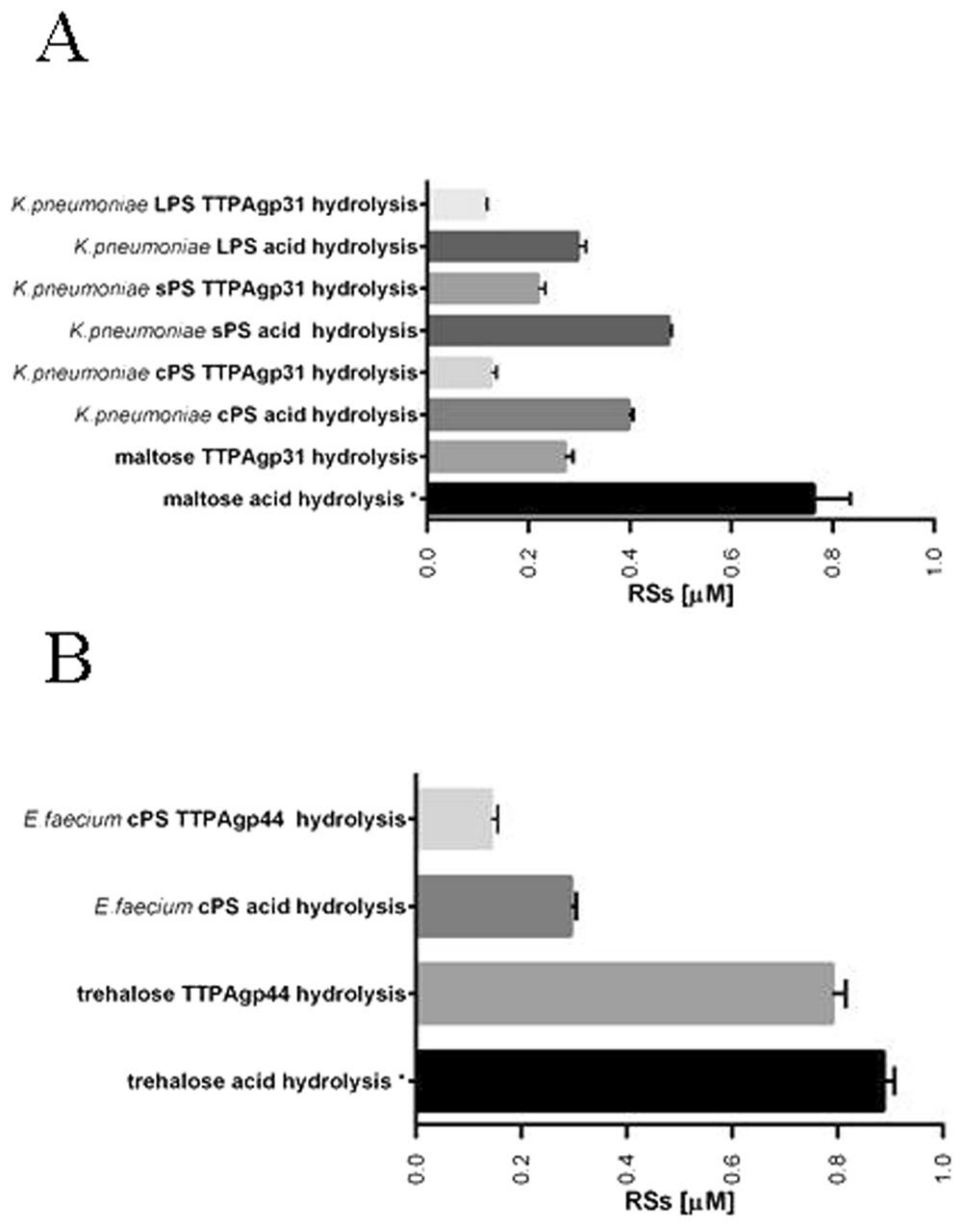
The hydrolytic activity of TTPAs toward disaccharide and PSs substrates. TTPAgp31 activity toward maltose and *K*. *pneumoniae* PCM 2713 PSs. *The given results are without the background of the negative control. TTPAgp44 activity toward trehalose and *E*. *faecium* PCM 1859 cPS. All results are presented as averages of results from three independent replicates in three parallel trials. Error bars represent the means, standard deviations (GraphPad Prism version 5.00 for Windows, GraphPad Software, San Diego California USA, (www.graphpad.com)).

The hydrolytic activity of the TTPAs was first performed towards disaccharides: α-lactose, β-lactose, trehalose, melibiose, cellobiose and maltose. Only maltose was hydrolyzed by TTPAgp31, while TTPAgp44 hydrolyzed trehalose. In the test conditions, TTPAgp31 released 35% of RSs with regards to the whole number of RSs obtained after acid hydrolysis (positive control). TTPAgp44 hydrolyzed trehalose with the efficiency of 87%. The hydrolyzing activity of the TTPAs towards disaccharides is shown in Fig. 3.

Based on bioinformatics analysis, both proteins carry peptidoglycan hydrolase domain with the probability of 40%. To check their activity towards peptidoglycan pNP-GlcNAc as well as resorufin-labeled casein, chromogenic substrates were used. No activity of both TTPAs towards both substrates was observed.

**Figure 4:**
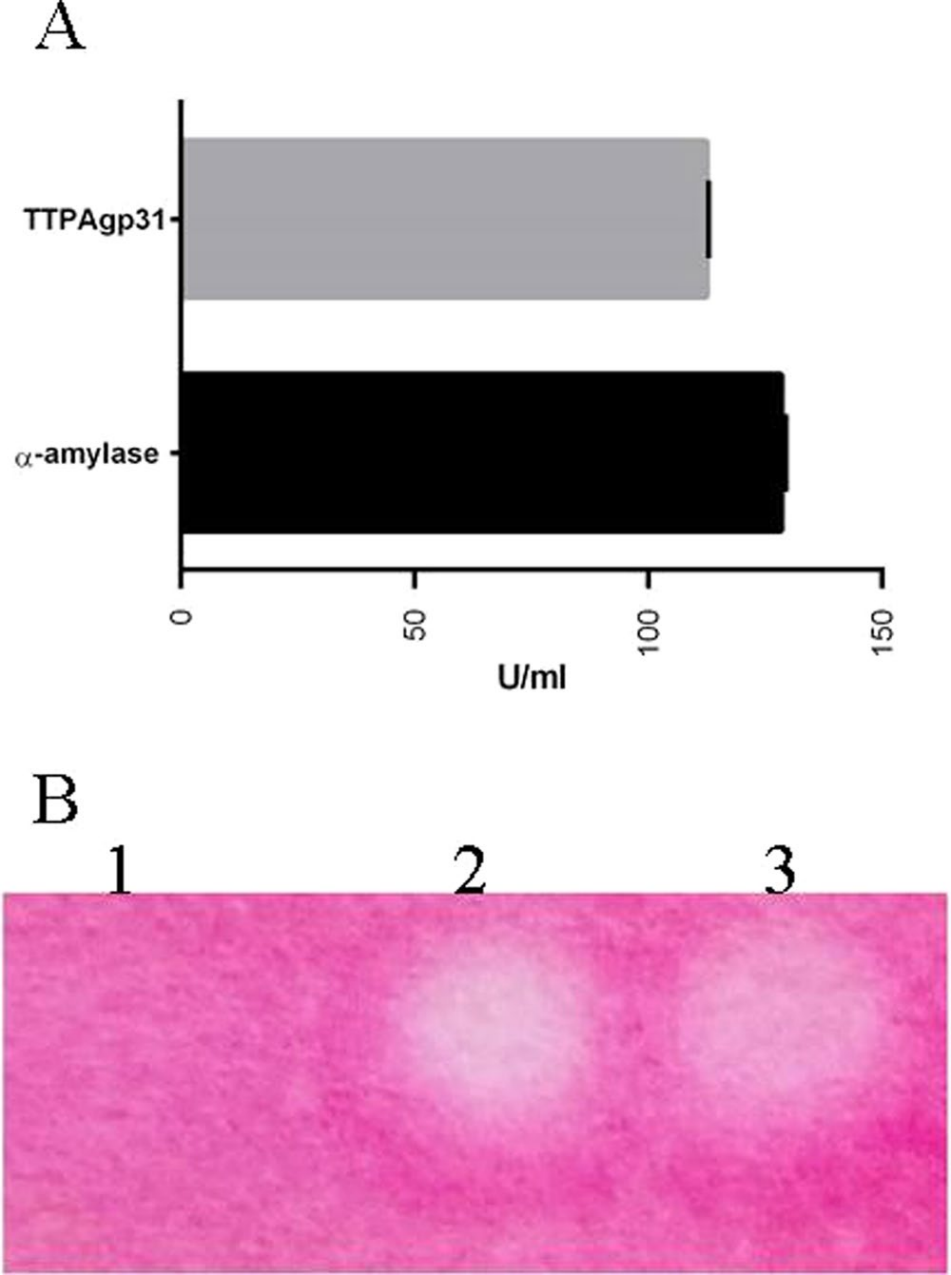
Hydrolytic activity of TTPAgp31 towards Red-starch. The activity of TTPAgp31 was compared to the *B*. *subtilis* α-amylase (positive control). The calculation was performed based on a protocol for assay of Alpha-amylase using Red-starch from Megazyme. All results are presented as averages of results from three independent replicates in three parallel trials. Error bars represent the means, standard deviations (GraphPad Prism version 5.00 for Windows, GraphPad Software, San Diego California USA, (www.graphpad.com)). Red-starch saturated filter paper treated with (3) TTPAgp31, (2) *B*. *subtilis* α-amylase, positive control, (1) phosphate buffer, the negative control.

Red-starch was tested as a next chromogenic substrate. Only TTPAgp31 was active against the polysaccharide showing almost as high activity as the α-amylase used in the assay as a positive control. The activity of the *B*. *subtilis* α-amylase (positive control) towards the Red-starch was calculated as 128.6 milli-Ceralpha U/ml, while TTPAgp31 as 112.8 milli-Ceralpha U/ml. It means that 1 µM of TTPAgp31 possess over 47 U of an amylase activity per 1 milliliter of the solution. The calculation was performed based on a protocol for assay of α-amylase using Red-starch from Megazyme^22^. Results of the experiment on Red-starch paper are presented in Fig. 4.

The overlay test on the agar plates showed that TTPAgp44 was significantly active towards *E*. *faecium* (PCM 1859) strain. It means that TTPAgp44 possess antibacterial activity towards other bacterial strain than TTPAgp31 reported before^19^.

To check the hydrolyzing activity of both proteins against the bacterial polysaccharides (PS), cPS, sPS and LPS from the *K*. *pneumoniae* and cPS from the *E*. *faecium* strains, were isolated.

The acid hydrolysis has been performed to obtain the total amount of the RSs of the bacterial PSs. The RSs calculation was made using Nelson-Somogoi assay^23^. The RSs concentration for *K*. *pneumoniae* capsular and the slime PSs (100 μg of dry mass), as well as for LPS was calculated as 0.39 μM ml^−1^, 0.47 μM ml^−1^ and 0.29 μM ml^−1^, respectively. After the *K*. *pneumoniae* PS hydrolysis using TTPAgp31 the RSs in the samples were 0.12, 0.21 and 0.11 μM ml^−1^ for cPS, sPS and LPS, respectively. The calculation of the RSs release has been performed considering the RSs present in the control sample lacking TTPAgp31.

The hydrolytic activity of TTPAgp44 was tested towards cPS of *E*. *faecium*. The RSs amount released after incubation with the protein was 0.143 μM ml^−1^ and reached almost 50% of the total RSs obtained when the same amount of the PSs was subjected to acid hydrolysis (positive control). No such release was observed in the control sample lacking the phage protein.

## Discussion

Bacteriophages are attractive and very effective antibacterial agents. It has been shown that phages degrade PSs and allow access to bacterial cell^24–27^. It is well-known that *K*. *pneumoniae* phages produce PS hydrolases which are associated with the tail spike and form characteristic halo zones in a spot agar plate assay^28^. In our studies, phage tail proteins named TTPAs belonging to KP32 and KP34 phages were tested in the screening assays on agar plates towards randomly selected bacterial strains, however, most of them have been isolated from human body and fluids. Their enzymatic activity displayed as the translucent halo zones creating on the bacteria lawns. The zones were not clear plaques meaning that these proteins didn’t lyse the bacterial cells, but destroyed the components localized outside the cells. In consequence, the translucent, but not clear zones were observed^24^. Interestingly, the proteins were active towards different bacterial strains. TTPAgp31 was active against *K*. *pneumoniae* (PCM 2713), *S*. *aureus* PCM 519 and *E*. *faecalis* PCM 2673, while TTPAgp44′s activity was observed towards *E*. *faecium* PCM 1859, *P*. *aeruginosa* PCM 2710 and *B*. *subtilis* PCM 2021. The antibacterial activity has been confirmed in antibiofilm assay. We used trimethyl tetrazolium chloride (TTC) assay that is versatile, high-throughput method of biofilm measure, applicable to a broad range of microorganisms^29^.

As we can see, although these proteins are classified to the same family of tail tubular protein A and have similar molecular masses they differ with respect to their physico-chemical features (such as Ip mentioned above) and the substrate specificity. That is in agreement with the previous observation that phages proteins have also different host specificities^30^. TTPAgp31 belongs to KP32 bacteriophage that is *Kp32virus* in the subfamily *Autographivirinae* (http://www.ictv.global/proposals-16/2016.023a-dB.A.v1.Kp32virus.pdf) and TTPAgp44 belongs to KP34 phage (*Kp34likevirus* in the subfamily *Autographivirinae*)^30^. Both phages come from *Podoviridae* family and are characterized as *Klebsiella pneumoniae* bacteriophages^30,31^. It must be also emphasized, that the substrate specificity of the proteins as separate molecules may differ to the specificity of the proteins which are combined together forming phage tail, by extension to the specificity of the bacteriophage particles.

In this paper, the hydrolytic activity of two TTPAs towards different saccharides substrates has been shown. In our previous paper, we have discussed the glucanase activity of TTPAgp31 that can be related to the presence of lectin-like domain and peptidoglycan hydrolase domain^19^. Despite low amino acid sequence homology between TTPAgp31 and TTPAgp44 (21%) the structural homology can be high as predicted by Phyre2 analysis (88% with 100.0% confidence) and both potential hydrolytic domains are present in TTPAgp44 as well. pNp-GlcNAc and resorufin-labeled casein as substrates were used to verify the N-acetyl-β-D-glucosaminidase and peptidase activity, respectively. These two enzymes are involved in peptidoglycan (PG) degradation, however, the PG hydrolysis is not limited only to those two enzymes (Moak and Molineux, 2004)^32^. Therefore, even if both TTPAs were not active against above substrates they should not be discriminated as PG hydrolases. PG consists of cross-linked amino acids and sugars which form alternating disaccharide of amino sugars: N-acetylglucosamine (NAG) and N-acetylmuramic acid (NAM). N-acetyl-β-D-glucosaminidase cleaves the N-acetylglucosaminyl-β-1,4-N-acetylmuramine bond, N-acetyl-β-D-muramidase (also termed lysozyme) cleaves N-acetylmuramyl-β-1,4-N-acetylglucosamine bond and N-acetylmuramyl-L-alanine amidase can hydrolyze the amide bond between the sugar and the peptide moieties. Endopeptidase hydrolyzes any of the peptide bonds between amino acids. Finally, the recently discovered γ-D-glutaminyl-L-lysine endopeptidase cleaves the gamma bond between D-glutamine and L-lysine residues^33^.

In the next experiments, disaccharides and Red-starch (according to Megazyme the substrate for α-amylase) have been used as substrates. TTPAgp31 hydrolyzed maltose and starch (better results were obtained at pH = 5.5–6.0 than at 8.0) suggesting the similar activity to α-glucosidase (EC.3.2.1.20) in terms of substrate specificity and pH range. α-Glucosidases are typical amylolytic hydrolases cutting the α-1,4 bonds releasing single glucose residue from the non-reducing end of substrates (oligosaccharides or polysaccharides). However, these enzymes are diverse in substrate specificity, optimum temperature reaction and transglucosylation activities^34^. α-Amylases belong to glycoside hydrolases (GH) number 13 and contain the catalytic site and subsides, which interact with single glucose residue in the substrate^35^. In general, differences in substrate specificity of the enzymes are explained by the difference in numbers of the subsites, a different affinity for glucose residues of each subsite, different amino acid composition and the differences in the catalytic^35^.

TTPAgp44 hydrolyzed trehalose breaking down the α,α-1,1-glycosidic bond that is characteristic for α,α-trehalase (glucohydrolase) (EC 3.2.1.28).

Previously^19^, for TTPAgp31 we have suggested the motif of D-X-D and/or D-X-E responsible for enzymatic activity of glycosyl hydrolases^36^. Our analysis let us suppose that the similar catalytic motif, which is D116-X-E118, is present in TTPAgp44.

The results of the hydrolytic activity tests towards *K*. *pneumoniae* PSs, TTPAgp31 hydrolyzed the capsular, slime PSs and the LPS. TTPAgp44 was active toward cPS of *E*. *faecium*. PSs of above bacteria usually contain mannose, galactose and glucose as predominant carbohydrate components which are linked via glycosidic bond and that are potential targets for TTPAs^37,38^. However, the substrate specificity depends on many factors, for example, a tertiary structure of the PSs^39^. In general, bacterial extracellular PSs can comprise homopolysaccharides and heteropolysaccharides synthesized at the cell membrane through processes involving nucleoside diphosphate sugars and isoprenoid lipid intermediates. The other group comprises levans and dextrans, formed essentially by extracellular processes without nucleoside diphosphate sugars or lipid intermediates, but dependent on the presence of specific substrates such as sucrose and closely related oligosaccharides^39^. Moreover, to understand the broad specificity of the proteins, we must emphasize that the TTPAs are the secondary phage adhesins and they come into action after a host recognition proses. Being a part of the phage tail, they are not naturally exposed to recognize the bacteria, unlike in the experiments described in this paper.

## Conclusions

Bacteriophage tail tubular proteins A displaying enzymatic activity, are not well known as dual-function proteins. They have been considered only as structural proteins and they enzymatic features were not studied. In our previous paper^19^ and here we present the results of our research aimed to explain the biological function of TTPAs based on two proteins, TTPAgp31 and TTPAgp44.

Our research showed that both proteins have a high structural homology, but different substrate specificity. TTPAgp31 has been identified as α-1,4-glucosidase which hydrolyzes maltose as well as starch. TTPAgp44 possesses trehalase-like activity. As it is known, hydrolases are very diverse in terms of their molecular mass and specificity^40^ and almost 16 different combinations of the catalytic domains of these enzymes have been documented^18^.

But our studies let us suggest that both proteins contain D-X-E motif localized in the β-stranded region which is essential for catalytic activity of glycoside hydrolases.

Our results showed also that TTPAs might be useful in the treatment of infections caused by biofilm-forming bacteria. TTPAgp31 is very effective against clinical isolates of *E*. *faecalis*, *P*. *aeruginosa* and *K*. *pneumoniae* while TTPAgp44 is effective against *P*. *aeruginosa*, *B*. *subtilis* and thermoresistant *E*. *faecium* strain which is a very dangerous foodborne pathogen. It must be emphasiezed that both proteins possess antibacterial activity against different bacterial strains which means that they have different antibacterial specificity.

Our results show that the phage proteins being separate molecules in experimental conditions may possess not obvious enzymatic activity and for that reason, they should be litigious examined.

## Materials and Methods

### Bacterial strains

All of the bacterial strains used in the experiments were obtained from the Polish Collection of Microorganisms (PCM) of the Institute of Immunology and Experimental Therapy, Polish Academy of Sciences (Wroclaw, Poland), namely: *Klebsiella pneumoniae* (PCM 1; PCM 2713), *Shigella flexneri* (PCM 2336), *Escherichia coli* (PCM 172; PCM 185; PCM 195; PCM 2711), *Proteus mirabilis* (PCM 543), *Citrobacter freundii* (PCM 1562), *Enterobacter aerogenes* (PCM 532), *Hafnia alvei* (PCM 1223); *Pseudomonas aeruginosa* (PCM 499, PCM 2710), *Enterococcus faecium* (PCM 1858; PCM 1859), *Enterococcus faecalis* (PCM 2673), *Bacillus subtilis* (PCM 2021) and *Staphylococcus aureus* (PCM 502; PCM 2054, PCM 519). The strains were stored at −80 °C and cultivated in Luria-Bertani (LB) broth medium (Difco). Bacteria were cultured at 37 °C stationary or with shaking.

### Cloning procedure

The genomes of phage KP32 and KP34 are deposited in the genomic database (GenBank): GQ413937 and GQ413938, respectively. TTPA encoding genes were selected for overexpression. Bacteriophage genes were obtained using polymerase chain reaction (PCR) with the following primers: TTPA from KP32 FW – GGATCCCATATGAACATGCAAGATGCTTAC, RV – GAATTCAAAGCTTACGACCGATGAGACCCT, TTPA from KP34 FW – GGATCCCATATGAGAGAACTTGATGCAATT, RV – GAATTCAAAGCTTAATACCATAAAACGAGCGCG.

The DNA of the bacteriophages was prepared as previously described^41^. PCR reactions were conducted using a two-phase program. The first phase consisted of seven and the second phase of 23 cycles. Taq polymerase (Fermentas) was used and the extension times for each gene were appropriate to gene length, min. 30 seconds to max. 2 minutes. Annealing temperature in the first phase was 48–52 °C and in the second phase 55–65 °C.

PCR products were cloned into the pGEM T-easy vector (T-vector, Promega) using T4 ligase. Constructs were transformed into *E*. *coli* DH5α bacteria using the heat-shock method and sequenced. Correct sequences were recloned into pET28a (Promega) expression vectors to obtain the phage tail proteins with an N-terminal six-histidine tag. Plasmid transformation into the competent *E*. *coli* BL21(DE3)plysS (Promega) cells was done using the heat shock method.

### Expression and purification of phage tail proteins

Bacteriophage proteins were expressed in *E*. *coli* BL21(DE3)plysS strain (Promega). Bacterial clones were propagated in LB broth (37 °C with shaking) with kanamycin and chloramphenicol to reach OD600 = 0.8. Protein expression was performed using 0.05 mmol l^−1^ IPTG (Roche) as an inductor and followed by overnight incubation at 9 °C. Cells were pelleted and suspended in 50 mmol l^−1^ Tris/HCl pH = 8.0 lysis buffer containing 0.2 mol l^−1^ NaCl supplemented with protease inhibitor cocktail tablets (Roche). Cells were sonicated 8 times for 30 seconds with 1 minute breaks. After debris removal via centrifugation (14000 × g for 50 minutes) supernatant was mixed with Ni^2+^-agarose beads and incubated at 37 °C for 1 hour on a rotary shaker. After batch chromatography, the beads were washed using lysis buffer to remove unbound proteins. Bounded proteins were eluted using lysis buffer containing 250 mmol l^−1^ imidazole. Imidazole was removed from the protein’s solution via dialysis on centrifugal filters containing membrane (Millipore) with a cutoff of 3 kDa. The concentration of protein was determined using the BCA method described by Smith *et al*.^42^ and eluted fractions were analyzed by SDS-PAGE using 12.5% gels according to the method of Laemmli *et al*.^43^.

### Agar overlay test

The hydrolytic activity of the phage proteins was determined using the spot assay described by Adams and Park (1956)^24^. The overnight cultures of bacteria were diluted to OD = 0.2 and pipetted onto agar plates. 10 µl of 47 µM of the proteins were spotted on the bacterial lawns and incubated at 37 °C overnight. The nutrient agar (pH = 7.2) composition was: beef extract (10 g) peptone (10 g) NaCl (5 g) and agar (20 g) suspended in 1000 ml and sterilized at 121 °C for 20 minutes.

### Glycolytic hydrolase activity measurement towards saccharide substrates and bacterial PSs

The hydrolytic activity of the two proteins has been first demonstrated towards the disaccharide substrates such as: α-lactose, β-lactose, trehalose, melibiose, cellobiose, maltose and saccharose. 0.1 ml of disaccharide solution (5 mg/ml) was incubated with 0.1 ml of protein solution (47 µM dissolved in 50 mmol l^−1^acetate buffer, pH = 5.5) for 1 h at 37 °C on a rotary shaker.

Slime polysaccharide was precipitated using 3 volumes of 96% ethanol (4 °C, overnight) from the supernatant obtained after centrifugation of the one-day bacterial culture. The precipitated sPS was centrifuged (14000 × g for 30 minutes at 4 °C) then dissolved in water, dialyzed against water, frozen and lyophilized. The capsular polysaccharide was extracted from the freeze-dried bacterial mass using 10% trichloroacetic acid (TCA) according to the method described by Gorska-Frączek *et al*.^44^.

Lipopolysaccharide was extracted by hot phenol-water method^45^ with some modifications. 1 g of dry bacterial mass was suspended in the phenol-water mixture (25 ml 1:1 v/v). LPS was extracted at 65 °C for 15 minutes and next, after cooling to 5 °C it was pelleted by centrifugation at 2500 rpm for 15 minutes. The water fraction was dialyzed to water for three days, concentrated by ultrafiltration. In order to eliminate contaminating nucleic acids and proteins treatment with DNase, RNase and Proteinase K was performed. For this purpose, proteinase RNase (40 µg/mL) (Roche) and DNase (20 µg/mL) (Roche) in the presence of 1 µL/mL 20% MgSO4 were added to the mixture and kept at 37 °C overnight. Finally, the Proteinase K was added (100 µg/mL) (Roche) at 65 °C for 1 hour. To remove the contamination, the ultrafiltration was performed at 100 000 g for 6 hours. The pelleted LPS was suspended in miliQ water and the ultrafiltration was repeated 4 times. Final purified LPS product was lyophilized and stored at 4 °C.

The enzyme activity test was performed as follows: 60 μl of PS (0.1 mg ml^−1^) was mixed with 50 μl of the phage protein (47 µM dissolved in 50 mmol l^−1^acetate buffer, pH = 5.5). The reaction mixtures were incubated at 37 °C on a rotary shaker for 3 hours. The enzyme activity was evaluated using the reducing sugars (RSs) determination according to Nelson-Somogoi method (1952)^23^. The RSs amount was compared to the total amount of RSs released after PSs and the disaccharides acid hydrolysis (10 mol l^−1^ HCl, 85 °C, 25′). The negative control contained disaccharides/PS and buffer lacking the phage proteins. The acid hydrolysis was performed according to the method of Kubler-Kielb *et al*.^46^.

### Enzymatic activity measurements toward chromogenic substrates

p-Nitrophenyl-2-acetamino-2-deoxy-β-D-glucopyranoside (pNP-GlcNAc) (Sigma-Aldrich), RedCL-Amylose (Megazyme) and Red-Starch (Megazyme) were used as substrates for TTPAs. 0.1 g of pNP-GlcNAc was dissolved in 10 ml of 50 mM sodium citrate pH 4.6 supplemented with 1% BSA. TTPAs (2 mM) were mixed with the substrate and incubated at 37 °C for 1 hour. The reaction was stopped using 0.4 M glycine buffer pH 10.4.

RedCL-Amylose (0.2 g) was suspended in 10 ml of 100 mM Bis-Tris Buffer pH 7.0. TTPAs (50 µl of 47 µM) were mixed with the substrate and incubated at 37 °C for 1 hour. The reaction was stopped using 96% ethanol.

0.6% Red-starch was made up in phosphate buffer pH 6.8. TTPAs (50 µl of 47 µM) were mixed with the substrate (0.95 ml) and incubated at 37 °C for 1 hour. The reaction was stopped using 96% ethanol. As a positive control 50 µl of α-amylase from Bacillus subtilis (Sigma-Aldrich) was used in the concentration of 50 µM.

The assay was also performed on Whatman filter paper grade 1 according to the procedure described by Martin *et al*.^47^. The filter paper was saturated with the starch solution, dried and then, 10 µl of 47 µM of TTPAs and Bacillus subtilis α-amylase (positive control) and phosphate buffer (negative control) were spotted on the Red-Starch paper and incubated at 37 °C for 15 minutes. Positive results were characterized by a marked color change from dark pink to white. For each test above, positive and negative controls were included.

### Antibiofilm activity

One-day biofilm preparation: Bacteria were cultured overnight in 5 ml of LB broth at 37 °C with shaking. Overnight cultures were diluted to OD_600_ = 0.2 using fresh LB. 100 µl of bacteria suspension was inoculated into a 96-well plate (CytoOne) and incubated for 20 hours at 37 °C. After that time bacteria were removed and the plate was dried for 15 minutes up-side-down on a sterile paper towel. TTPAs were added (0.47 µM) and incubated at 37 °C for the next 24 hours. The next day, the OD_600_ was measured and after that 50 µl of 0.1% trimethyl tetrazolium chloride (TTC) was added to each well. After 1 hour of incubation at 37 °C the OD_540_ was measured on a Biotec microplate reader. Microtitre plate wells containing growth medium without any bacterial culture–sterility control; wells containing cell cultures but without TTPAs–control regarded as 100% cell mass. All trials were performed in triplicate and the mean value was calculated with the standard deviation range.
